# Supplementary material for: A ratiometric fluorescent probe for pH detection based on Ag2S quantum dots–carbon dots nanohybrids
Source: R Soc Open Sci. 2020 Jul 22;7(7):200482. doi: 10.1098/rsos.200482 (PMC7428231; doi:10.1098/rsos.200482)
Supplement: Supplementary data [file rsos200482supp1.doc]

Supporting Information for

A ratiometric fluorescent probe for pH detection based on Ag_2_S quantum dots - carbon dots nanohybrids

Xiaoxue Lei,† Yiying Fu,† Yuan Wu,* Lu Chen* and Jiangong Liang

College of Science, Huazhong Agricultural University, Wuhan 430070, P.R. China. E-mail:yuanwu@mail.hzau.edu.cn,chenlu@mail.hzau.edu.cn; Tel: +86-27-8728-3712; Fax: +86-27-8728-2133

† These authors contributed equally to this work.

**Supplementary Figures**

**
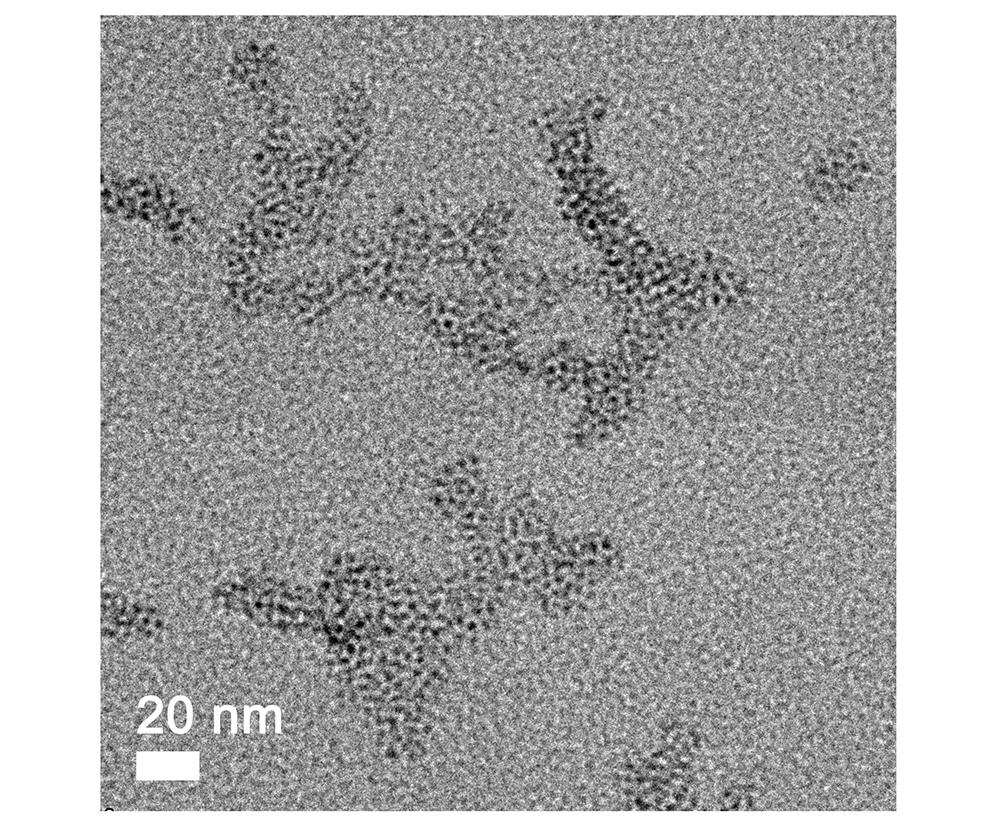
**

**Fig. S1** HR-TEM of Ag_2_S QDs.

**
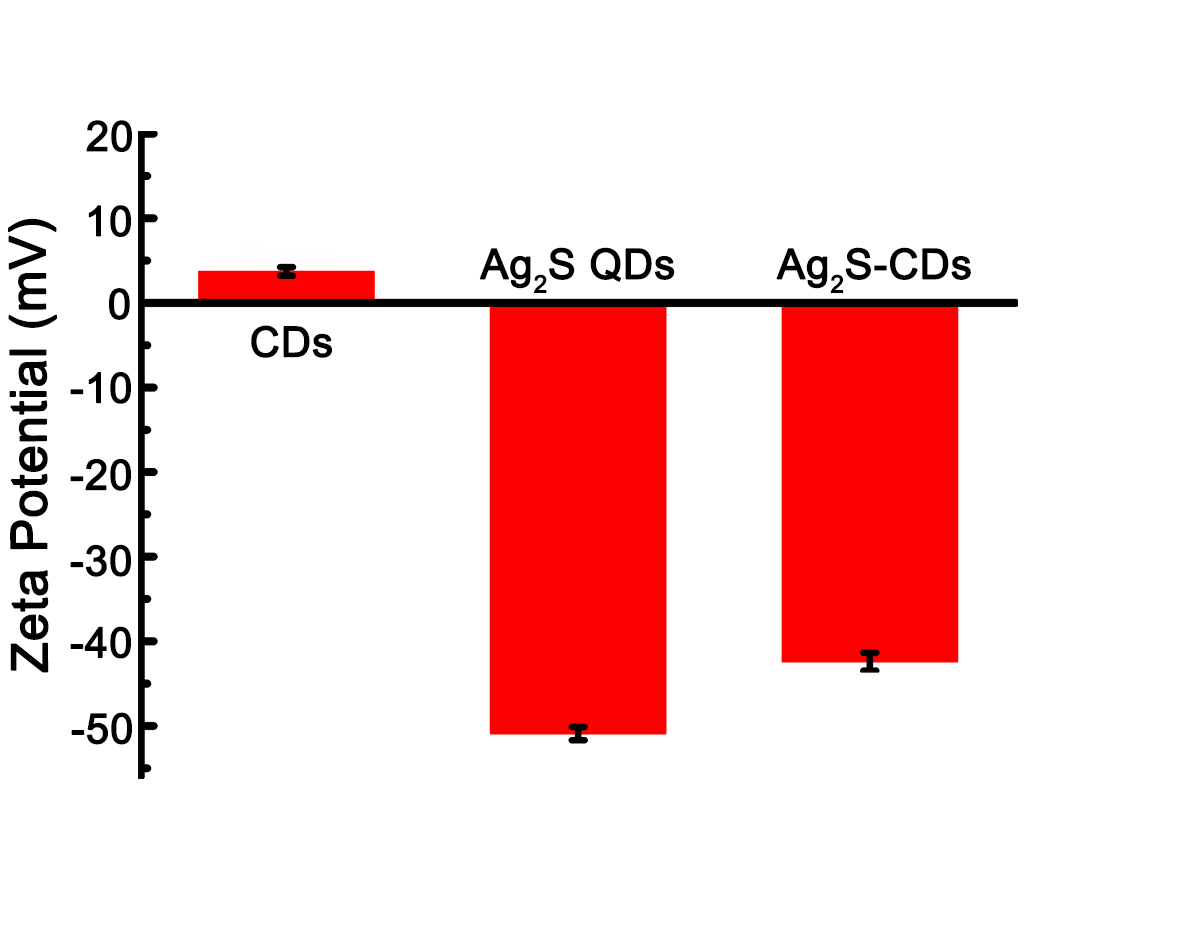
**

**Fig. S2** The Zeta potentials of CDs, Ag_2_S QDs and Ag_2_S-CDs.

**
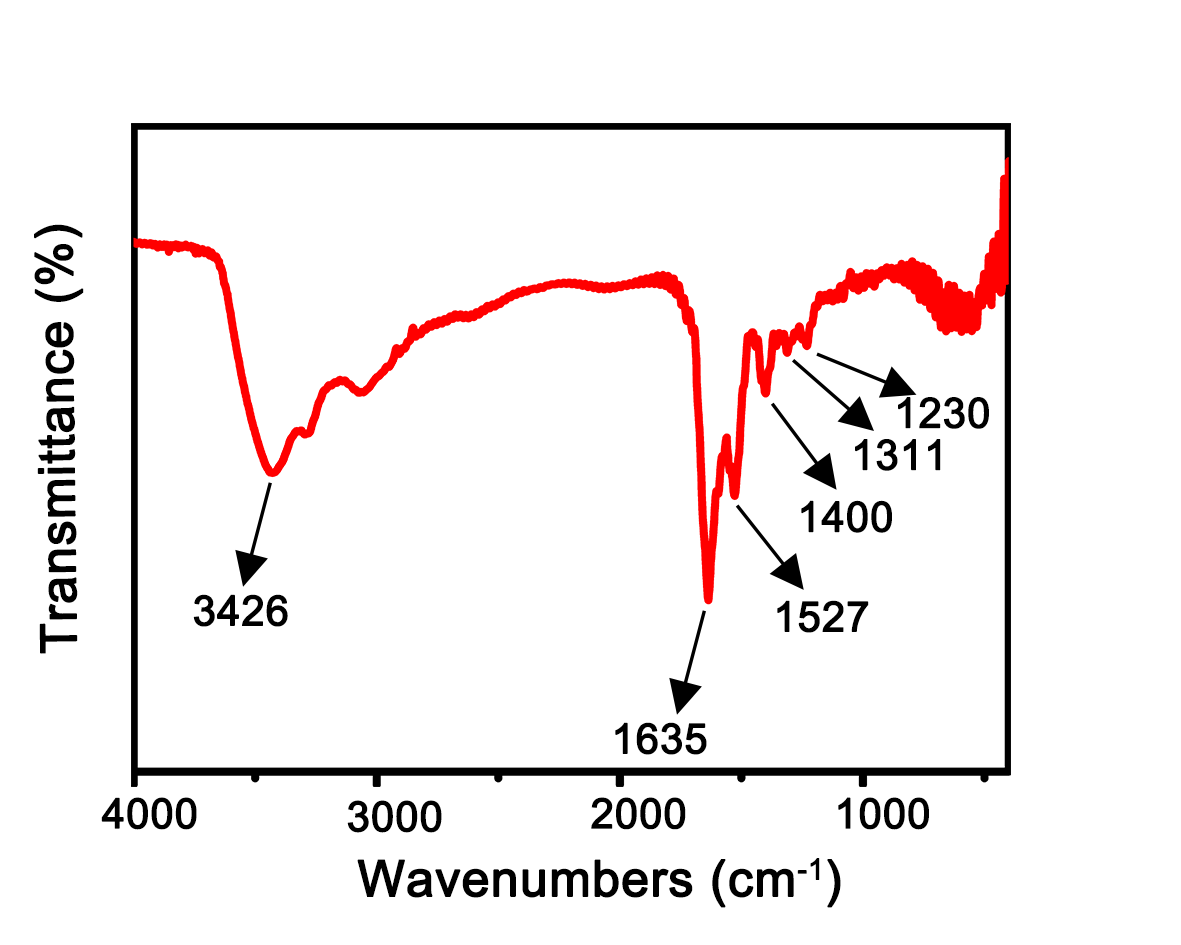
**

**Fig. S3** FTIR spectrum of Ag_2_S-CDs.

**
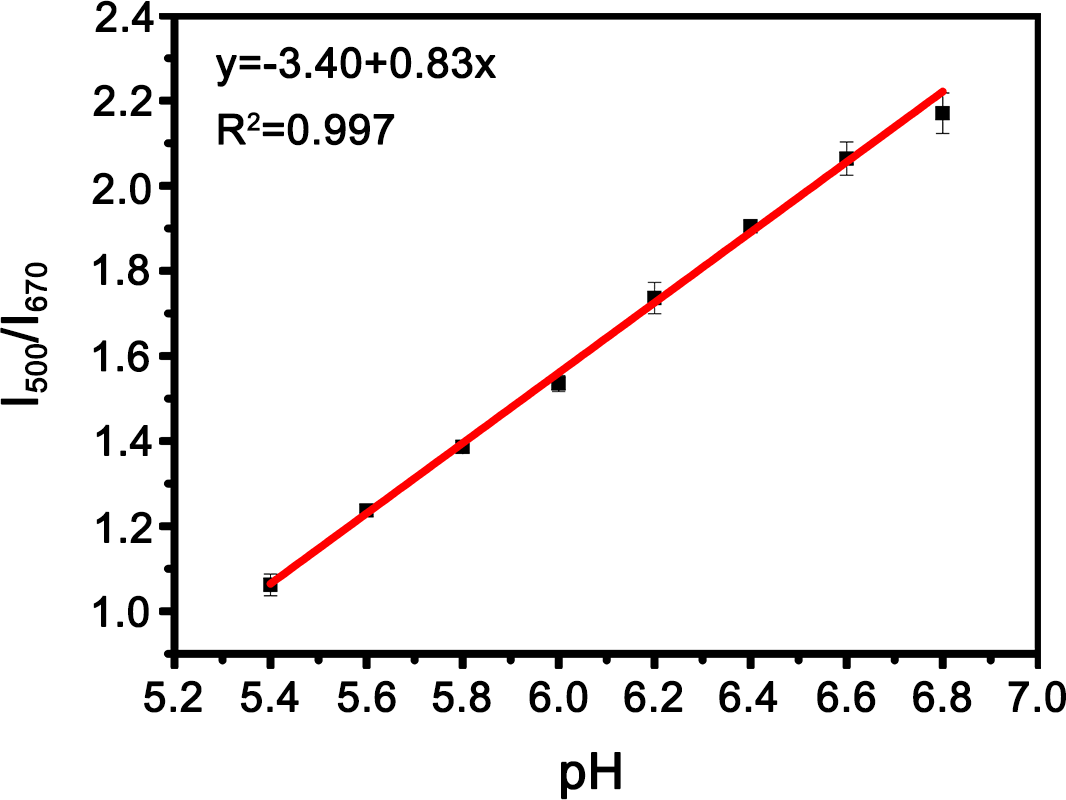
**

**Fig. S4** The I_500_/I_670_ ratios of Ag_2_S-CDs versus the pH values within the range of 5.4-6.8.
